# Generation of Unusual Aromatic Polyketides by Incorporation of Phenylamine Analogues into a C-Ring-Cleaved Angucyclinone

**DOI:** 10.3390/molecules26071959

**Published:** 2021-03-31

**Authors:** Hua Xiao, Guiyang Wang, Zhengdong Wang, Yi Kuang, Juan Song, Jing Jin, Min Ye, Donghui Yang, Ming Ma

**Affiliations:** State Key Laboratory of Natural and Biomimetic Drugs, School of Pharmaceutical Sciences, Peking University, 38 Xueyuan Road, Haidian District, Beijing 100191, China; 15521370039@163.com (H.X.); wangguiyang09@163.com (G.W.); zdwang@bjmu.edu.cn (Z.W.); kuangyi@bjmu.edu.cn (Y.K.); fltcsong@163.com (J.S.); jinjing@bjmu.edu.cn (J.J.); yemin@bjmu.edu.cn (M.Y.)

**Keywords:** angucyclinones, disubstituted phenylamines, nonenzymatic conversion, Nrf2 transcription activation

## Abstract

Angucyclinones are aromatic polyketides that possess impressive structural diversity and significant biological activities. The structural diversity of these natural products is attributed to various enzymatic or nonenzymatic modifications on their tetracyclic benz(*a*)anthracene skeleton. Previously, we discovered an unusual phenylamine-incorporated angucyclinone (**1**) from a marine *Streptomyces* sp. PKU-MA00218, and identified that it was produced from the nonenzymatic conversion of a C-ring-cleaved angucyclinone (**2**) with phenylamine. In this study, we tested the nonenzymatic conversion of **2** with more phenylamine analogues, to expand the utility of this feasible conversion in unusual angucyclinones generation. The (3-ethynyl)phenylamine and disubstituted analogues including (3,4-dimethyl)phenylamine, (3,4-methylenedioxy)phenylamine, and (4-bromo-3-methyl)phenylamine were used in the conversion of **2**, which was isolated from the fermentation of *Streptomyces* sp. PKU-MA00218. All four phenylamine analogues were incorporated into **2** efficiently under mild conditions, generating new compounds **3**–**6**. The activation of **3**–**6** on nuclear factor erythroid 2-related factor 2 (Nrf2) transcription were tested, which showed that **4** possessing a dimethyl-substitution gave most potent activity. These results evidenced that disubstitutions on phenylamine can be roughly tolerated in the nonenzymatic reactions with **2**, suggesting extended applications of more disubstituted phenylamines incorporation to generate new bioactive angucyclinones in the future.

## 1. Introduction

**Figure 1:**
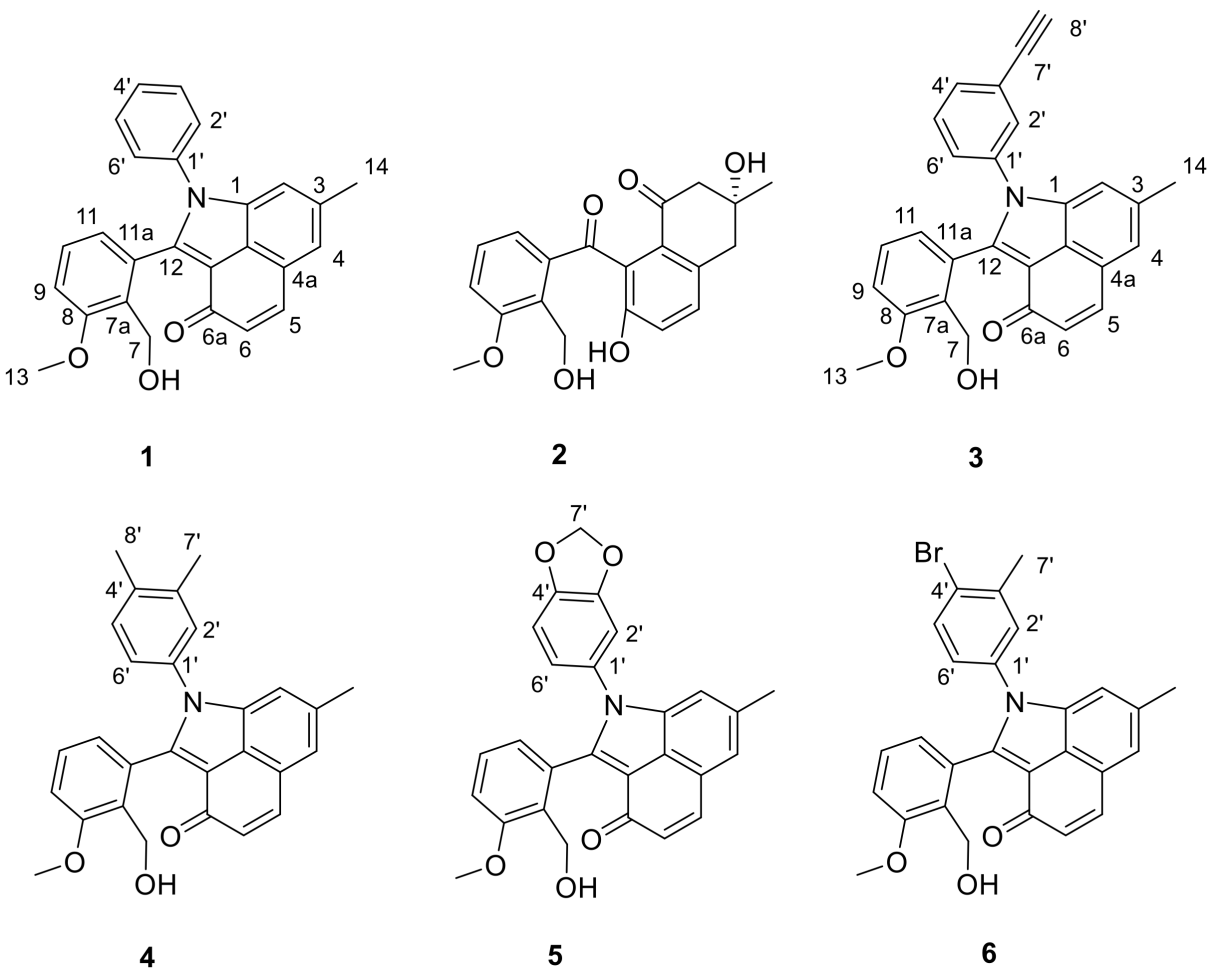
The structure of compounds **1**–**6**.

Angucyclinones are aromatic polyketides produced by type II polyketide synthases (PKSs) from actinomycetes exclusively [1]. The structures of these natural products feature a tetracyclic benz(*a*)anthracene skeleton, which can be modified by enzymatic or nonenzymatic reactions to achieve impressive structural diversity. As a part of our ongoing project for natural product discovery and biosynthesis from marine bacteria [2,3,4,5,6], we previously discovered an unusual phenylamine-incorporated angucyclinone (**1**) featuring 1-phenylbenzo(*cd*)indol-3(1*H*)-one moiety from a marine *Streptomyces* sp. PKU-MA00218 [2]. Based on genome sequencing, bioinformatics analysis, heterologous expression, gene deletion, and nonenzymatic conversion in vitro, we identified that **1** was produced from the nonenzymatic conversion of a C-ring-cleaved angucyclinone (**2**) with phenylamine, and generated a series of derivatives by the incorporation of monosubstituted phenylamine analogues into **2**. These derivatives represented a new group of angucyclinones and showed different degrees of activation activities on nuclear factor erythroid 2-related factor 2 (Nrf2) transcriptions in HepG2 cells, highlighting the powerful combination of biosynthesis and nonenzymatic reactions in the generation of structurally unusual and bioactive molecules. To further expand the structural diversity of this group of phenylamine analogues-incorporated angucyclinones, we report herein the generation of derivative **3** by the incorporation of (3-ethynyl)phenylamine, and **4**–**6** by the incorporation of disubstituted phenylamines into **2** (Figure 1). The generation of **3**–**6** can be efficiently achieved under mild conditions, and the activation of **3**–**6** on Nrf2 transcriptions were subsequently tested.

## 2. Results and Discussion

### 2.1. Generation of ***3**–**6*** by Nonenzymatic Incorporation of Phenylamine Analogues into C-Ring-Cleaved Angucyclinone ***2***

**Scheme 1:**
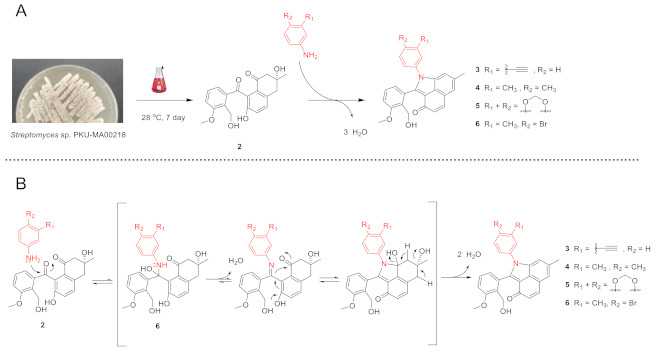
The conversion of **2** to **3**–**6** and proposed conversion mechanism. (**A**) The conversion of **2** to **3**–**6**. (**B**) Proposed conversion mechanism of **2** to **3**–**6**.

Monosubstituted phenylamine analogues have been efficiently incorporated into **2** under mild conditions, previously [2]. To test the efficiency of other phenylamine analogues in the incorporation, we selected four analogues with distinct substitutions on the phenylamine. The (3-ethynyl)phenylamine contains an ethynyl moiety that is never used previously and may facilitate the biological targets identification through a click reaction with chemical probes; the (3,4-dimethyl)phenylamine, (3,4-methylenedioxy)phenylamine and (4-bromo-3-methyl)phenylamine contain groups that affect the electron density of phenyl moiety in different ways. The four phenylamine analogues were individually incubated with **2**, which was isolated with a combination of chromatographic methods from a large-scale fermentation of *Streptomyces* sp. PKU-MA00218 (Materials and Methods). Four new angucyclinone products **3**–**6** were efficiently produced under the similar mild conditions used previously (Scheme 1A and Supplementary Materials). The conversion mechanisms of **2** to **3**–**6** were similarly proposed as that of **2** to **1** (Scheme 1B) [2]. Compounds **3**–**6** were purified by semipreparative HPLC, and their structures were elucidated with UV, IR, NMR, and high resolution electrospray ionization mass spectroscopy (HR-ESI-MS) analyses (Supplementary Materials).

### 2.2. Structural Elucidation of ***3**–**6***

**Figure 2:**
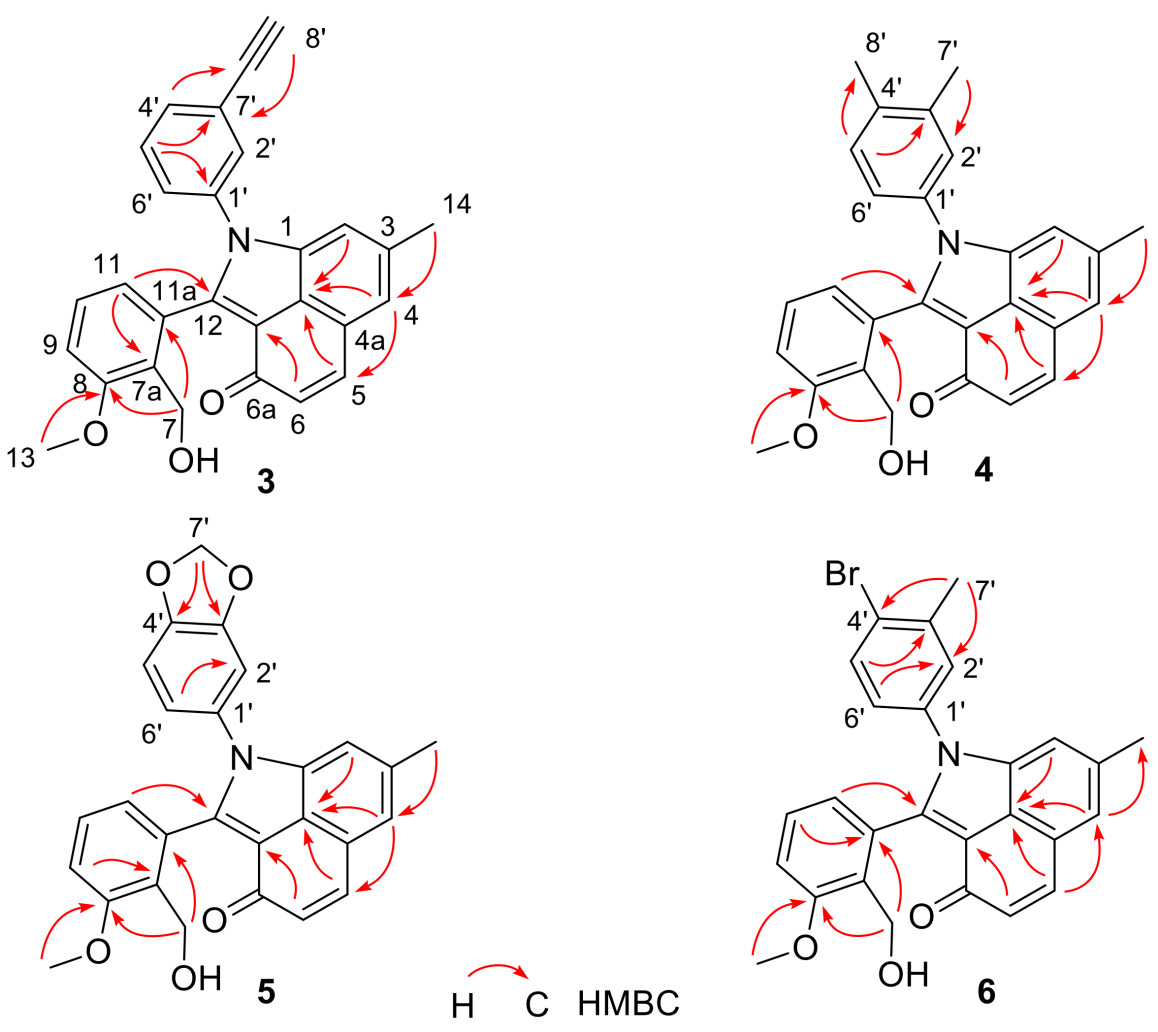
Key HMBC correlations of compounds **3**–**6**.

Compound **3** was obtained as green-yellow solid, UV (MeOH) *λ*_max_ (log *ε*): 272 (5.17), 358 (5.13), 415 (5.06) nm. IR (KBr) *ν*_max_: 3287, 2919, 1636, 1594, 1582, 1460, 1296, 1263, 1028 cm^−1^. HR-ESI-MS analysis afforded an [M + H]^+^ ion at *m/z* 420.1600 (calcd for C_28_H_22_NO_3_, 420.1594) giving the molecular formula of **3** as C_28_H_21_NO_3_. ^1^H-NMR (600 MHz, DMSO-*d*_6_) and ^13^C-NMR (150 MHz, DMSO-*d*_6_) data, Table 1. The ^1^H-NMR and ^13^C-NMR spectra of **3** resembled that of **1**, except that the resonances at *δ*_H_ 7.54 (H-2′/H-6′), 7.45 (H-3′/H-5′), 7.41 (H-4′) and *δ*_C_ 136.0 (C-1′), 127.6 (C-2′/C-6′), 129.3 (C-3′/C-5′), 128.3 (C-4′) in **1** were replaced by *δ*_H_ 7.70 (H-2′), 7.50 (H-4′), 7.45 (H-5′), 7.29 (H-6′) and *δ*_C_ 136.4 (C-1′), 130.7 (C-2′), 122.7 (C-3′), 131.8 (C-4′), 129.7 (C-5′), 128.6 (C-6′) in **3**. Besides, compound **3** contains additional signals attributed to an ethynyl moiety at *δ*_H_ 4.31 (s, H-8′) and *δ*_C_ 82.4 (C-7′), 82.2 (C-8′). These results suggested the difference between **3** and **1** was that **3** contained a (3-ethynyl)phenylamino moiety instead of a phenylamino moiety. The (3-ethynyl)phenylamino moiety in **3** was further confirmed by HMBC experiment (Figure 2 and Supplementary Materials) and comparison of ^1^H-NMR and ^13^C-NMR data with published data of (3-ethynyl)phenylamino moiety in literatures [7,8]. Thus, compound **3** was identified as a new (3-ethynyl)phenylamine-incorporated angucyclinone analogue.

Compound **4** was obtained as green–yellow solid, UV (MeOH) *λ*_max_ (log *ε*): 271 (5.99), 359 (6.02), 415 (5.99) nm. IR (KBr) *ν*_max_: 3375, 2919, 1635, 1596, 1504, 1458, 1055, 1028, 1007, 821, 759 cm^−1^. HR-ESI-MS analysis afforded an [M + H]^+^ ion at *m/z* 424.1907 (calcd for C_28_H_26_NO_3_, 424.1907) giving the molecular formula of **4** as C_28_H_25_NO_3_. ^1^H-NMR (600 MHz, DMSO-*d*_6_) data, Table 1; ^13^C-NMR (150 MHz, DMSO-*d*_6_) data, Table 1. The ^1^H-NMR and ^13^C-NMR spectra of **4** resembled that of **1**, except that the resonances at *δ*_H_ 7.54 (H-2′/H-6′), 7.45 (H-3′/H-5′), 7.41 (H-4′) and *δ*_C_ 136.0 (C-1′), 127.6 (C-2′/C-6′), 129.3 (C-3′/C-5′), 128.3 (C-4′) in **1** were replaced by *δ*_H_ 7.36 (H-2′), 7.16 (H-5′), 7.19 (H-6′) and *δ*_C_ 137.4 (C-1′), 128.3 (C-2′), 136.7 (C-3′), 133.6 (C-4′), 130.1 (C-5′), 124.9 (C-6′) as well as two additional methyl groups signals at *δ*_H_ 2.28 (s, 3H, H-7′), 2.21 (s, 3H, H-8′) and *δ*_C_ 19.4 (C-7′), 19.0 (C-8′) in **4**. These results suggested the only difference between **4** and **1** was that **4** contained a (3,4-dimethyl)phenylamino moiety instead of a phenylamino moiety. The (3,4-dimethyl)phenylamino moiety in **4** was further confirmed by HMBC experiment (Figure 2 and Supplementary Materials) and comparison of ^1^H-NMR and ^13^C-NMR signals with published data of (3,4-dimethyl)phenylamino moiety in literature [9]. Thus, compound **4** was identified as a new (3,4-dimethyl)phenylamine-incorporated angucyclinone analogue.

Compound **5** was obtained as green-yellow solid, UV (MeOH) *λ*_max_ (log *ε*): 273 (5.99), 355 (5.98), 415 (5.95) nm. IR (KBr) *ν*_max_: 3346, 2917, 1635, 1596, 1502, 1489, 1460, 1263, 1239, 1029 cm^−1^. HR-ESI-MS analysis afforded an [M + H]^+^ ion at *m/z* 440.1490 (calcd for C_27_H_22_NO_5_, 440.1492) giving the molecular formula of **5** as C_27_H_21_NO_5_. ^1^H-NMR (600 MHz, DMSO-*d*_6_) and ^13^C-NMR (150 MHz, DMSO-*d*_6_) data, Table 1. The ^1^H-NMR and ^13^C-NMR spectra of **5** resembled that of **1**, except that the resonances at *δ*_H_ 7.54 (H-2′/H-6′), 7.45 (H-3′/H-5′), 7.41 (H-4′) and *δ*_C_ 136.0 (C-1′), 127.6 (C-2′/C-6′), 129.3 (C-3′/C-5′), 128.3 (C-4′) in **1** were replaced by *δ*_H_ 7.18 (H-2′), 7.04 (H-5′), 6.98 (H-6′) and *δ*_C_ 129.7 (C-1′), 108.2 (C-2′), 147.1 (C-3′), 147.5 (C-4′), 108.7 (C-5′), 121.6 (C-6′) as well as additional methylenedioxy signals at *δ*_H_ 6.10 (br s, 1H, H-7′a), 6.08 (br s, 1H, H-7′b), and *δ*_C_ 101.9 (C-7′) in **5**. These results suggested the only difference between **5** and **1** was that **5** contained a (3,4-methylenedioxy)phenylamino moiety instead of a phenylamino moiety. The (3,4-methylenedioxy)phenylamino moiety in **5** was further confirmed by HMBC experiment (Figure 2 and Supplementary Materials) and comparison of ^1^H-NMR and ^13^C-NMR signals with published data of (3,4-methylenedioxy)phenylamino moiety in literatures [10,11]. Thus, compound **5** was identified as a new (3,4-methylenedioxy)phenylamine-incorporated angucyclinone analogue.

Compound **6** was obtained as green-yellow solid, UV (MeOH) *λ*_max_ (log *ε*): 271 (6.27), 356 (6.26), 415 (6.20) nm. IR (KBr) *ν*_max_: 3343, 2917, 2850, 1635, 1584, 1477, 1463, 1296, 1263, 1152, 1053, 1029 cm^−1^. HR-ESI-MS analysis afforded an [M + H]^+^ ion at *m/z* 488.0862 (calcd for C_27_H_23_BrNO_3_, 488.0856) giving the molecular formula of **6** as C_27_H_22_BrNO_3_. The ^1^H NMR (600 MHz, DMSO-*d*_6_) and ^13^C NMR (150 MHz, DMSO-*d*_6_) data, Table 1. The ^1^H-NMR and ^13^C-NMR spectra of **6** resembled that of **1**, except that the resonances at *δ*_H_ 7.54 (H-2′/H-6′), 7.45 (H-3′/H-5′), 7.41 (H-4′) and *δ*_C_ 136.0 (C-1′), 127.6 (C-2′/C-6′), 129.3 (C-3′/C-5′), 128.3 (C-4′) in **1** were replaced by *δ*_H_ 7.61 (H-2′), 7.62 (H-5′), 7.28 (H-6′) and *δ*_C_ 135.5 (C-1′), 130.1 (C-2′), 138.6 (C-3′), 124.0 (C-4′), 132.8 (C-5′), 127.0 (C-6′) as well as an additional methyl group signals at *δ*_H_ 2.32 (s, 3H, H-7′), and *δ*_C_ 22.5 (C-7′) in **6**. These results suggested the difference between **6** and **1** was that **6** contained a (4-bromo-3-methyl)phenylamino moiety instead of a phenylamino moiety. The (4-bromo-3-methyl)phenylamino moiety in **6** was further confirmed by HMBC experiment (Figure 2 and Supplementary Materials) and comparison of ^1^H-NMR and ^13^C-NMR signals with published data of (4-bromo-3-methyl)phenylamino moiety in literatures [12,13]. Thus, compound **6** was identified as a new (4-bromo-3-methyl)phenylamine-incorporated angucyclinone analogue.

### 2.3. Biological Activity Assays

**Figure 3:**
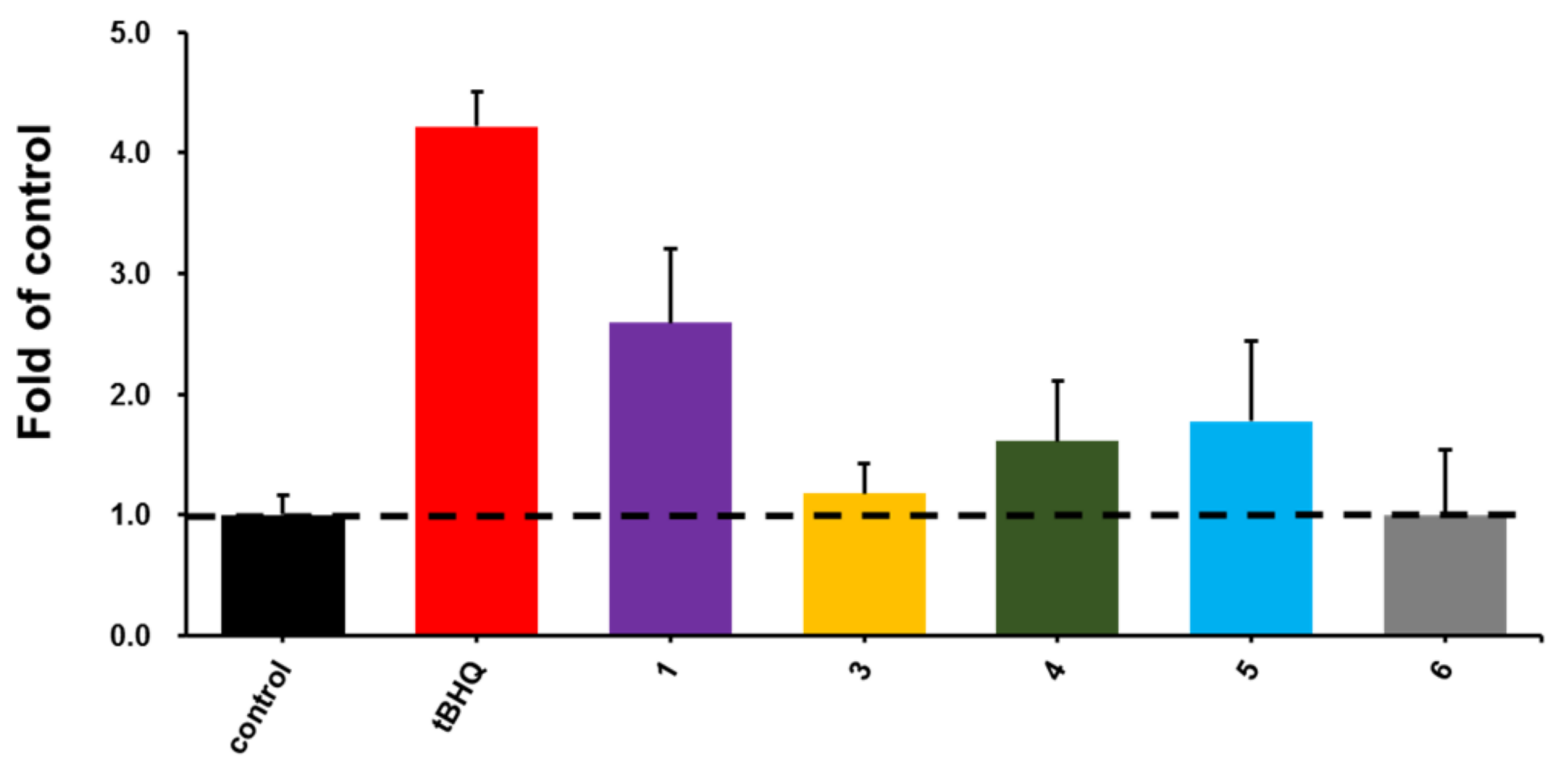
The activation activities on Nrf2 transcription of compounds **1** and **3**–**6** at 10 μM, with tertiary butylhydroquinone (tBHQ) as the positive control. The activation potency of compounds were shown by the folds of the transcription activation level when cells were treated with DMSO.

According to previous procedures, the activation of **3**–**6** on nuclear factor erythroid 2-related factor 2 (Nrf2) transcription in HepG2 cells was evaluated by using a luciferase reporter assay with tertiary butylhydroquinone (tBHQ) as the positive control [14]. Compound **3**–**5** showed Nrf2 transcription activation levels 1.18, 1.61, and 1.78 folds to that of the DMSO-treated negative control (Figure 3). Compound **1** showed better activity than **3**–**5**, suggesting the substitutions on the phenylamine moieties in **3**–**5** impair the Nrf2 transcription activation. More assays need to be carried out to assess the potential biological activities of these unusual angucyclinones.

## 3. Materials and Methods

### 3.1. General Experimental Procedures

Optical rotations were measured on an Autopol III automatic polarimeter (Rudolph Research Analytical, Hackettstown, NJ, USA). UV spectra were collected on a NanoDrop 2000C spectrophotometer (Thermo Scientific, Waltham, MA, USA). IR spectra were recorded with a NICOLET iS50 FT-IR (Thermo Scientific). ^1^H and ^13^C-NMR spectra were collected on a Bruker Avance-600 NMR spectrometer (Bruker Corporation, Billerica, MA, USA). HR-ESI-MS spectra were collected on a Waters Xevo G2 Q-TOF spectrometer equipped with an ESI ion source (Waters, Milford, MA, USA). The mass instrument was operated in positive ion mode with a capillary potential at 3000 V and sampling cone potential at 20 V. The *m*/*z* range was recorded from 105 to 1000 Da, sample concentration was between 0.005 and 0.05 mg/mL, and all mass spectra were externally calibrated using a sodium formate solution. HPLC analysis was performed on an Agilent 1260 series (Agilent Technologies, Santa Clara, CA, USA) with a C_18_ RP-column (Eclipse XDB C_18_, 250 × 4.6 mm, 5 μm, Agilent Technologies, Santa Clara, CA, USA). Semi-preparative HPLC was performed on a SSI 23201 system (Scientific Systems Inc., State College, PA, USA) with a YMC Pack ODS-A column (250 × 10 mm, 5 μm, YMC Co., LTD. Shimogyo-ku, Kyoto, Japan). Medium performance liquid chromatography (MPLC) was performed on a LC 3000 series (Beijing Tong Heng Innovation Technology, Beijing, China) with a Claricep^TM^ Flash i-series C_18_ cartridge (20–35 μm, 40 g, Bonna-Agela, Wilmington, DE, USA). Size exclusion chromatography was carried out using a Sephadex LH-20 (GE Healthcare, Chicago, IL, USA) column. All fermentations were carried out in MQD-B1R shakers (Minquan Instrument Co., Ltd., Shanghai, China).

### 3.2. The Fermentation and Isolation of ***2***

The spores of *Streptomyces* sp. PKU-MA00218 was inoculated in 250 mL Erlenmeyer flasks containing 50 mL seed medium (yeast extract 1 g, peptone 5 g, beef extract 1 g, FePO_4_ 0.01 g, agar 18 g, and sea salts 33 g in 1.0 L distilled H_2_O, pH 7.4) at 28 °C, 220 rpm in a rotary shaker for three days. Then each of the seed cultures (16 mL) was inoculated into autoclaved 2 L Erlenmeyer flasks containing 400 mL of production medium (soluble starch 10 g, casein 0.3 g, KNO_3_ 2 g, K_2_HPO_4_∙3H_2_O 2 g, MgSO_4_∙7H_2_O 0.05 g, CaCO_3_ 0.02 g, FeSO_4_∙7H_2_O 0.01 g, and sea salts 33 g in 1.0 L distilled H_2_O, pH 7.0). The fermentations (total 24 L) were continued at 28 °C, 220 rpm for seven days. The D101 resins (5 g/100 mL) were added 12 h before the fermentation finished.

The resins and cell mass were harvested by centrifugation and extracted with MeOH. The MeOH extract was concentrated, resuspended in H_2_O, and extracted with EtOAc for three times. The EtOAc extract was fractionated by MPLC, using a gradient elution program from 5% MeOH in H_2_O to 100% MeOH over 30 min, with the flowrate of 15 mL/min under the UV detection at 254 nm, to give 15 fractions (a1–a15). Fraction a10–a15 were combined and subjected to Sephadex LH-20 chromatography eluting with MeOH to give 5 fractions (b1–b5). Fraction b4 was further purified by semi-preparative HPLC eluting with CH_3_CN/H_2_O (45/55, *v/v*) with the flowrate of 2 mL/min under the UV detection at 254 nm, to afford compound **2** (16 mg).

### 3.3. The Conversion of ***2*** to ***3**–**6***

Each of the four phenylamine analogues was incubated with **2** (~3 mg) (molar ratio of **1** to phenylamine analogue is 1 to 10) in 500 μL CH_3_CN/HAc (4/1, *v/v*) at 50 °C for 3 h, respectively. The reaction products were purified by semi-preparative HPLC using CH_3_CN/H_2_O (60/40, *v/v*) as the mobile phase, with a flow rate of 2 mL/min with UV detection at 320 nm, to afford compound **3** (1.4 mg, yield: 59%), **4** (1.7 mg, yield: 71%), **5** (1.8 mg, yield: 73%), **6** (1.3 mg, yield: 47%).

### 3.4. Biological Activity Assays

The assay of Nrf2 transcription activation of **3**–**6** were carried out following the reported protocols [15]. Human hepatocellular carcinoma (HepG2) cells were stably transfected with Nrf2/ARE luciferase reporter to generate the HepG2-ARE-C8 cells. The HepG2-ARE-C8 cells were plated in 96-well plates and exposed with tested compounds at 10 μM, with tertiary butylhydroquinone (tBHQ) as the positive control and DMSO as the negative control. After 6 h treatment, the cells were harvested in the luciferase cell culture lysis reagent and used for luciferase activity determination. The luciferase activities were measured using the Luciferase Assay System (Promega, WI, USA) on a Centro LB 960 microplate luminometer (Berthold, Germany). The transcription activities of tested compounds were obtained in triplicates and showed as the folds of the transcription level when cells were treated with DMSO [16].

## 4. Conclusions

In this study, four unusual phenylamine analogues-incorporated angucyclinones **3**–**6** containing benzo(*cd*)indol-3(1*H*)-one moieties were efficiently generated, expanding the structural diversity of angucyclinones. The generation of **3**, which contains an ethynyl moiety, may facilitate the biological targets (not limited to Nrf2 transcription activation) identification through a click reaction with chemical probes in the future. The high efficient incorporation of the disubstituted phenylamine analogues in the generation of **4**–**6** further evidenced the high reactivity of **2** when reacting with phenylamine analogues, which may not be limited by substitution groups on the phenylamine. These results set the stage for the incorporation of more diverse phenylamine analogues, such as dihalogen-substituted phenylamines, to further expand the structural diversity of this unusual group of angucyclinones. The new introduced chemical groups can imbed new biological activities into the angucyclinone analogues, and more biological activities need to be assayed in our next steps.

## Figures and Tables

**Table 1:** The ^1^H-NMR (600 MHz) and ^13^C-NMR (150 MHz) data of compounds **1** and **3**–**6** in DMSO-*d*_6_.

NO.	1		3		4		5		6	
	*δ*_H_ (*J* in Hz)	*δ*_C_	*δ*_H_ (*J* in Hz)	*δ*_C_	*δ*_H_ (*J* in Hz)	*δ*_C_	*δ*_H_ (*J* in Hz)	*δ*_C_	*δ*_H_ (*J* in Hz)	*δ*_C_
1	-	134.8	-	134.9	-	135.0	-	135.2	-	134.8
2	7.27, s	114.0	7.25, s	113.9	7.23, s	114.2	7.26, s	114.1	7.30, s	114.1
3	-	134.1	-	134.3	-	134.0	-	134.0	-	134.3
4	7.56, s	124.1	7.55, s	124.2	7.54, s	124.1	7.54, s	124.0	7.55, s	124.2
4a	-	113.1	-	113.1	-	113.0	-	112.9	-	113.1
5	7.85, d (9.6)	137.2	7.85, d (9.6)	137.3	7.84, d (9.5)	137.2	7.84, d (9.5)	137.2	7.84, d (9.5)	137.3
6	6.53, d (9.6)	132.9	6.52, d (9.6)	132.9	6.52, d (9.5)	132.9	6.51, d (9.5)	132.9	6.52, d (9.5)	132.9
6a	-	180.2	-	180.2	-	180.2	-	180.1	-	180.2
7	4.70, d (11.5)	55.2	4.71, br d (9.7) ^2^	55.1	4.65, br d (11.0)	55.4	4.71, br d (11.0)	55.1	4.71, br d ^2^	55.1
	4.50, d (11.5)		4.47, dd (12.1, 7.2)	-	4.47, br dd (11.0, 4.0)	-	4.46, br dd (11.0, 4.6)	-	4.47, dd (12.7, 7.4)	-
7a	-	130.2	-	130.3	-	130.1	-	130.1	-	130.2
8	-	157.1	-	157.1	-	157.2	-	157.1	-	157.1
9	7.02, dd (8.0, 1.0)	111.9	7.02, d (8.0)	112.1	7.02, d (8.0)	111.9	7.04, d ^2^ (8.0)	112.0	7.03, d (8.5)	112.1
10	7.11, t (8.0)	127.9	7.13, t (8.0)	128.0	7.13, t (8.0)	128.0	7.17, t (8.0)	128.0	7.15, t (8.0)	128.1
11	6.61, dd (8.0, 1.0)	122.5	6.65, br d (7.6)	122.5	6.62, br d (8.0)	122.5	6.67, br d (8.0)	122.4	6.63, br d (7.6)	122.5
11a	-	130.9	-	130.8	-	131.1	-	131.1	-	130.9
12	-	145.8	-	145.8	-	145.9	-	146.0	-	145.6
12a	-	123.0	-	123.1	-	123.0	-	123.0	-	123.1
12b	-	123.3	-	123.3	-	123.3	-	123.2	-	123.3
13	3.85, s	55.5	3.84, s	55.6	3.84, s	55.6	3.85, s	55.6	3.84, s	55.6
14	2.51, s	21.5	2.51, s ^1^	21.5	2.51, s^1^	21.6	2.52, s ^1^	21.5	2.51, s ^1^	21.5
7-OH	4.68, br s		4.70, br s ^2^	-	4.62, br s	-	4.69, br s	-	4.70, br s ^2^	-
1′	-	136.0	-	136.4	-	137.4	-	129.7	-	135.5
2′	7.54, br d (8.3)	127.6	7.70, br s	130.7	7.36, br s	128.3	7.18, s ^2^	108.2	7.61, s ^2^	130.1
3′	7.45, br t (7.7)	129.3	-	122.7	-	136.7	-	147.1	-	138.6
4′	7.41, m	128.3	7.50, d (8.0)	131.8	-	133.6	-	147.5	-	124.0
5′	7.45, br t (7.7)	129.3	7.45, m	129.7	7.16, br s ^2^	130.1	7.04, d ^2^	108.7	7.62, d ^2^	132.8
6′	7.54, br d (8.3)	127.6	7.29, m	128.6	7.19, br s ^2^	124.9	6.98, br s	121.6	7.28, m	127.0
7′	-		-	82.4	2.28, s	19.4	6.10, br s	101.9	2.32, s	22.5
							6.08, br s			
8′	-		4.31, s	82.2	2.21, s	19.0	-	-	-	-

^1^ signals were overlapped with DMSO. ^2^ signals were overlapped with others.

## Data Availability

Not applicable.

## Supplementary Materials

The following are available online. Figure S1: HPLC analysis of reactions of the phenylamine analogues with **2**. Figures S2–S6: NMR, HR-ESI-MS, and IR spectra of compound **3**, Figures S7–S11: NMR, HRESIMS, and IR spectra of compound **4**, Figures S12–S16: NMR, HRESIMS, and IR spectra of compound **5**, Figures S17–S21: NMR, HRESIMS, and IR spectra of compound **6**.
