# Supplementary material for: Generation of Unusual Aromatic Polyketides by Incorporation of Phenylamine Analogues into a C-Ring-Cleaved Angucyclinone
Source: Molecules. 2021 Mar 31;26(7):1959. doi: 10.3390/molecules26071959 (PMC8038006; doi:10.3390/molecules26071959)

Generation of Unusual Angucyclinones by Incorporation of Phenylamine Analogues into a Biosynthetic Intermediate

Hua Xiao [1,†], Guiyang Wang [1,†], Zhengdong Wang [1], Yi Kuang [1], Juan Song [1], Jing Jin [1], Min Ye [1], Donghui Yang [1,*], and Ming Ma [1,*]

[1] State Key Laboratory of Natural and Biomimetic Drugs, School of Pharmaceutical Sciences, Peking University, 38 Xueyuan Road, Haidian District, Beijing 100191

[†]These authors contributed equally

* Correspondence: ydhui@bjmu.edu.cn (D.Y.); mma@bjmu.edu.cn (M.M.)

Supplmentary Materials

Figure S1. HPLC analysis of reactions of the phenylamine analogues (**3a-6a**) with **2** to generate compounds **3-6**.. **S3**
Figure S2. The ^{1}H NMR (600 MHz) spectrum of **3** in DMSO-d_6…......................................**S4**
Figure S3. The ^{13}C NMR (150 MHz) spectrum of **3** in DMSO -d_6………....………….......**S5**
Figure S4. The HRESIMS spectrum of **3**..**S6**
Figure S5. The IR spectrum of **3**..**S7**
Figure S6. The ^{1}H NMR (600 MHz) spectrum of **4** in DMSO-d_6..**S8**
Figure S7. The ^{13}C NMR (150 MHz) spectrum of **4** in DMSO -d_6...**S9**
Figure S8. The HRESIMS spectrum of **4** **S10**
Figure S9. The IR spectrum of **4**..**S11**
Figure S10. The ^{1}H NMR (600 MHz) spectrum of **5** in DMSO-d_6......................................**S12**
Figure S11. The ^{13}C NMR (150 MHz) spectrum of **5** in DMSO-d_6.....................................**S13**
Figure S12. The HRESIMS spectrum of **5**...**S14**
Figure S13. The IR spectrum of **5**..**S15**
Figure S14. The ^{1}H NMR (600 MHz) spectrum of **6** in DMSO-d_6......................................**S16**
Figure S15. The ^{13}C NMR (150 MHz) spectrum of **6** in DMSO -d_6....................................**S17**
Figure S16. The HRESIMS spectrum of **6**...**S18**
Figure S17. The IR spectrum of **6**..**S19**

Figure S1. HPLC analysis of reactions of the phenylamine analogues (**3a**-**6a**) with **2** to generate compounds **3**-**6**. Lane I, the standard **2**; lane II, the reaction of (3-ethynyl)phenylamine (**3a**) with **2**; lane III, the reaction of (3,4-dimethyl)phenylamine (**4a**) with **2**; lane IV, the reaction of (3,4-methylenedioxy)phenylamine (**5a**) with **2**; lane V, the reaction of (4-bromo-3-methyl)phenylamine (**6a**) with **2**. Compound **2** was fully converted into **3**-**6** in each reaction. The compound labelled with asterisk was an unknown impurity in (3,4-methylenedioxy)phenylamine (**5a**) sample.

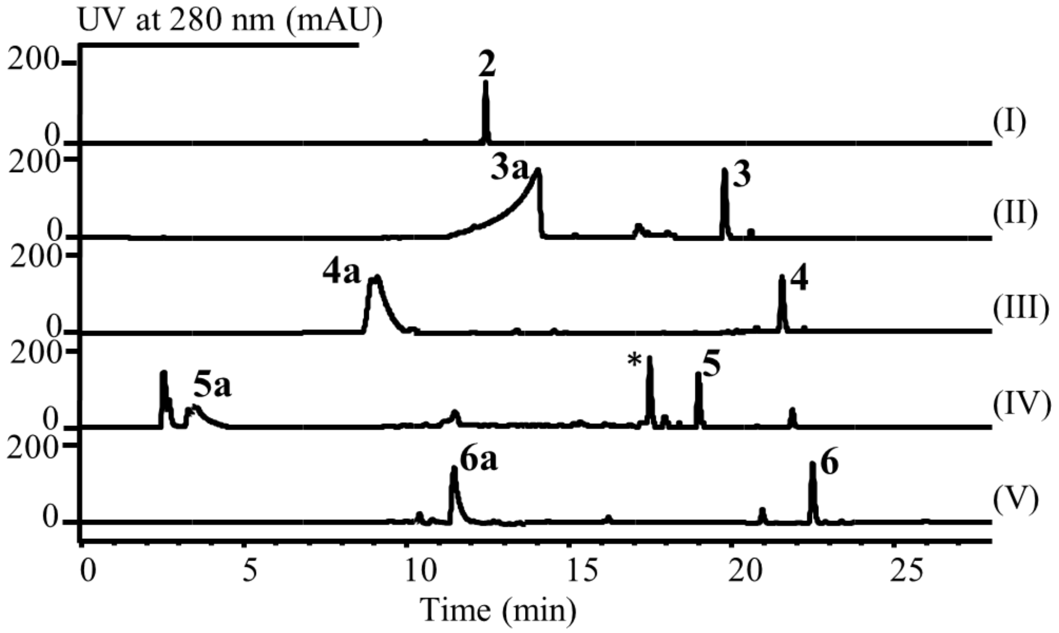

Figure S2. The ^{1}H NMR (600 MHz) spectrum of **3** in DMSO-d_6.

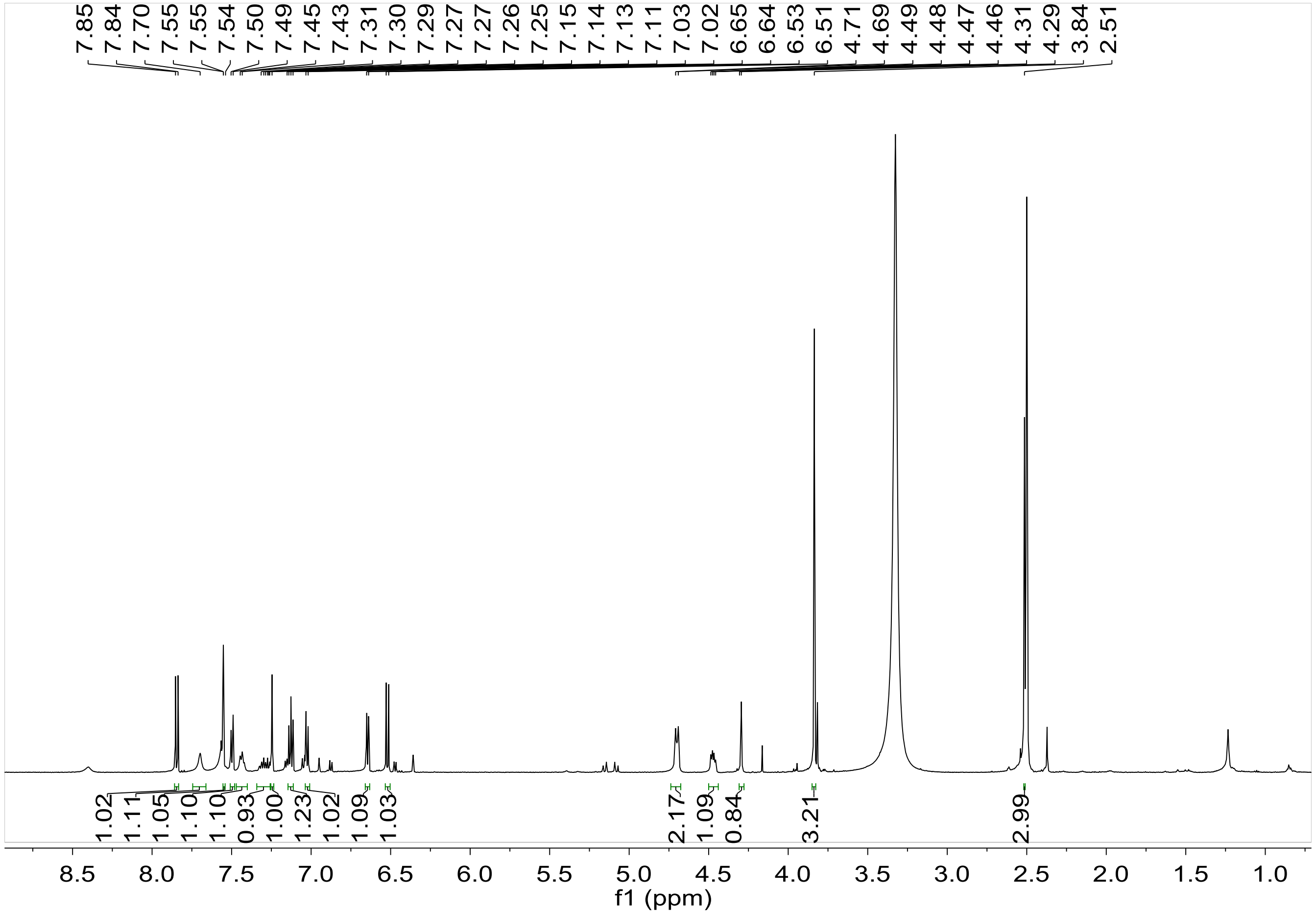

Figure S3. The ^{13}C NMR (150 MHz) spectrum of **3** in DMSO-d_6.

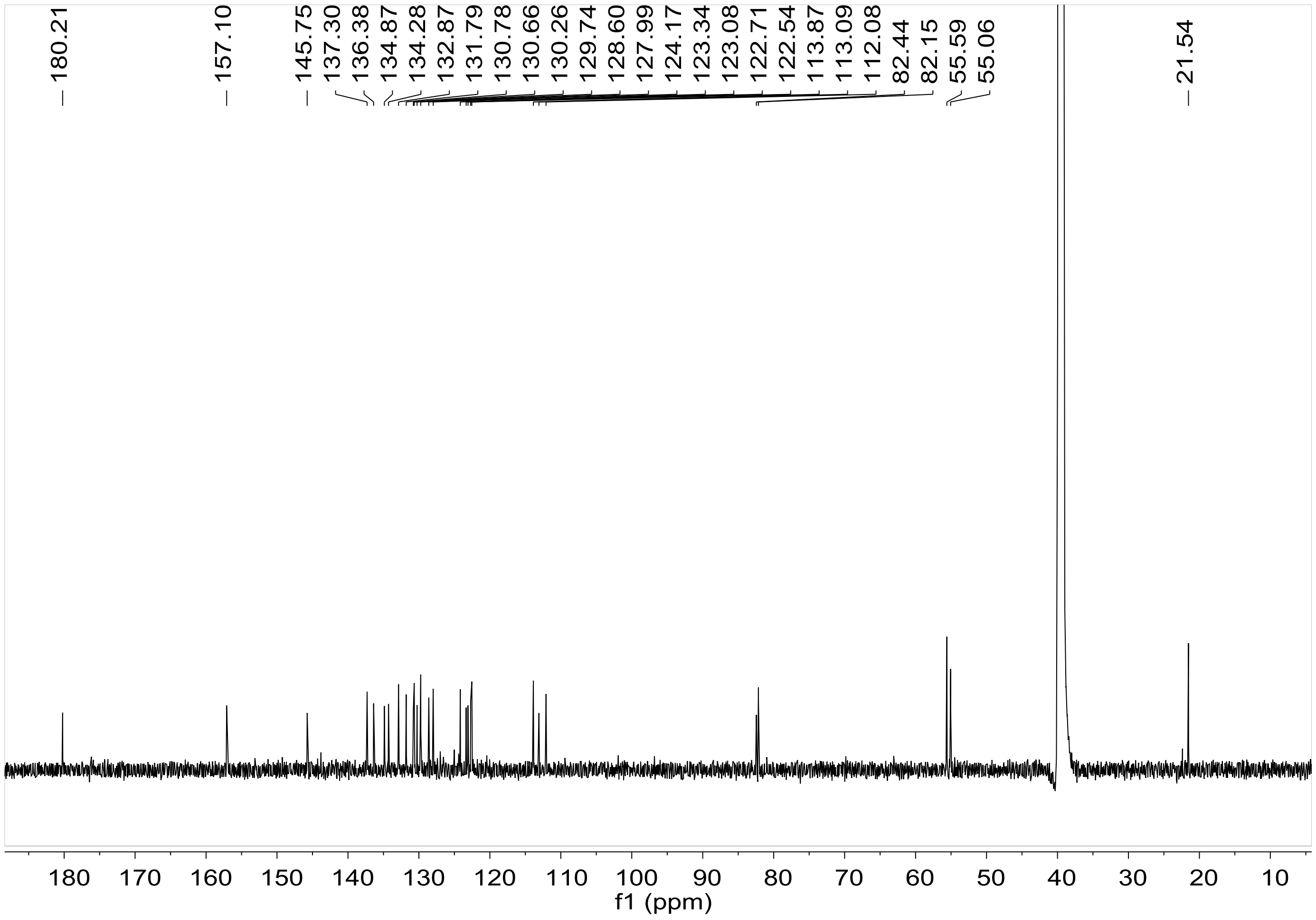

Figure S4. The HRESIMS spectrum of **3**.

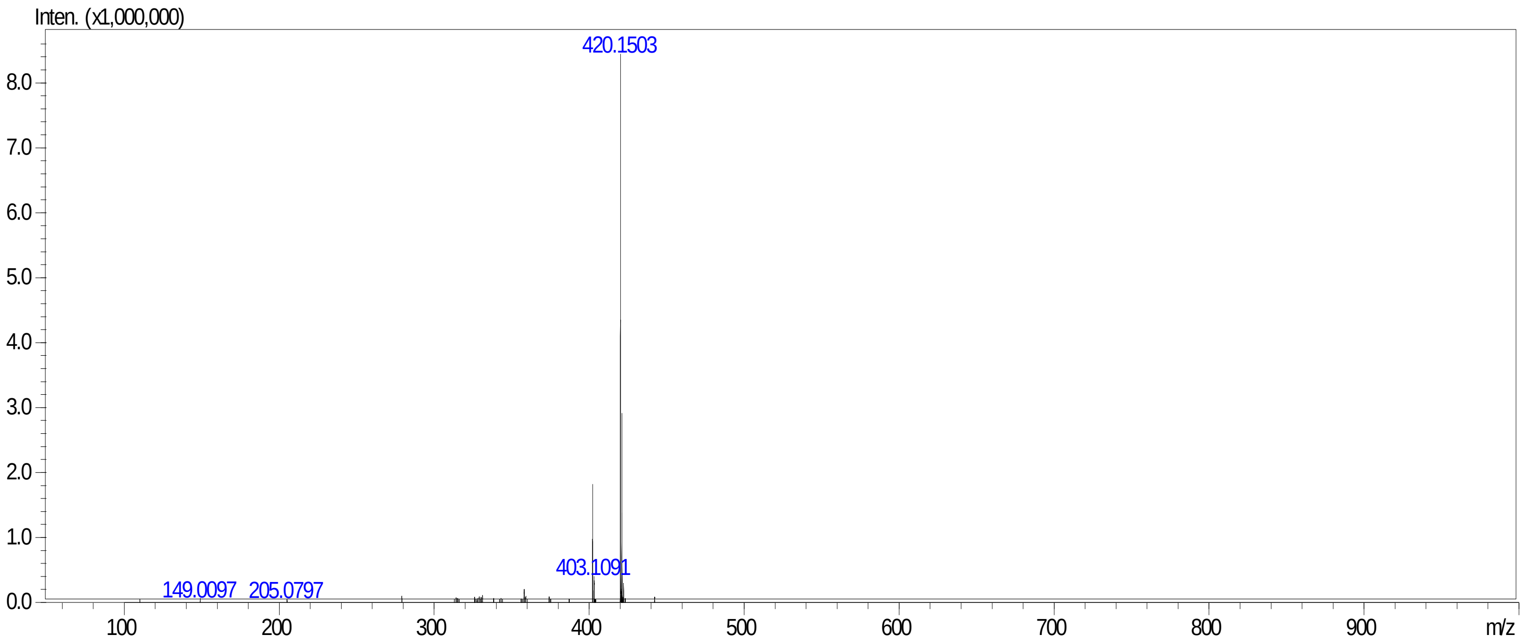

Figure S5. The IR spectrum of **3**.

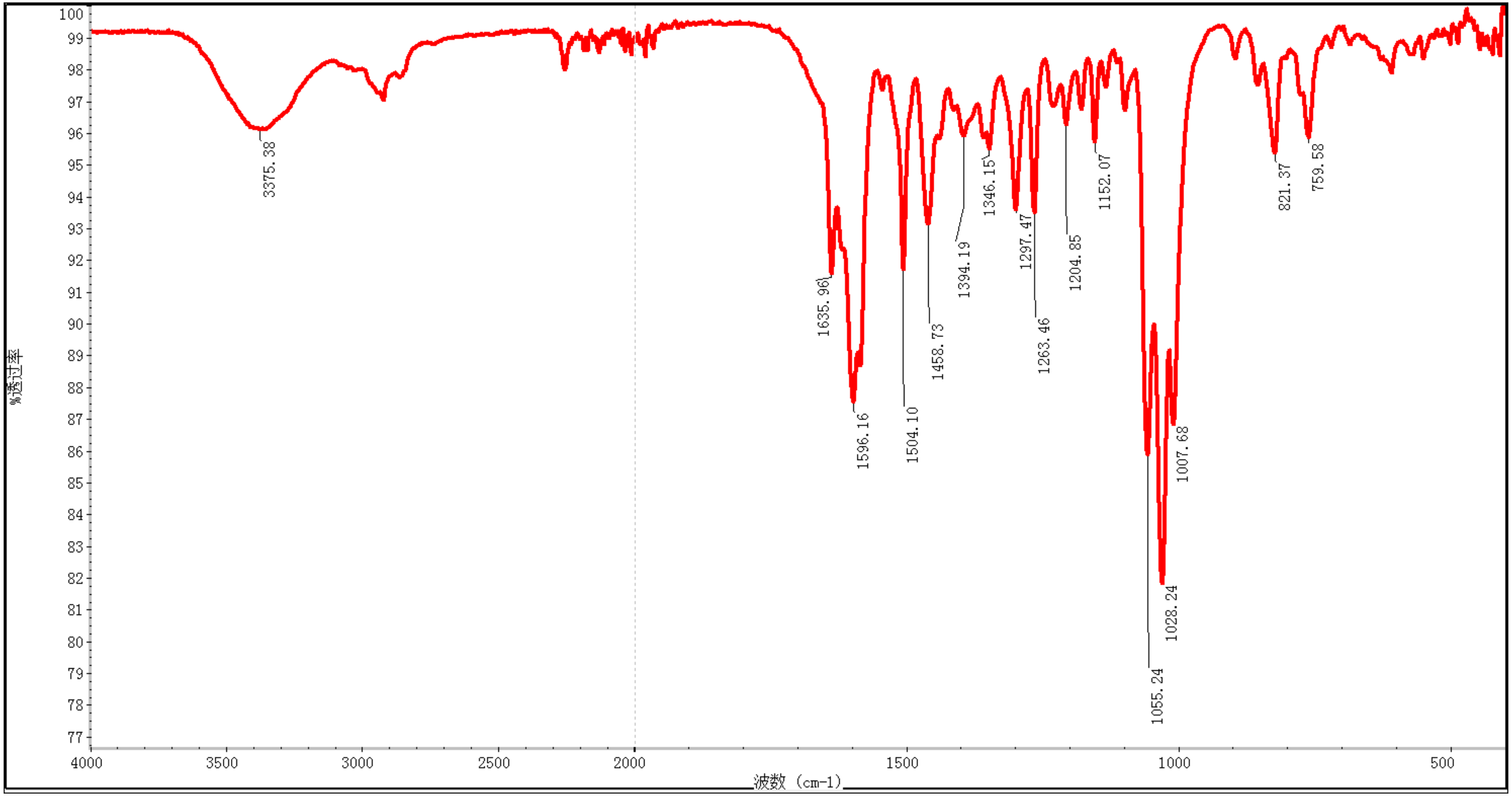

Figure S6. The ^{1}H NMR (600 MHz) spectrum of **4** in DMSO-d_6.

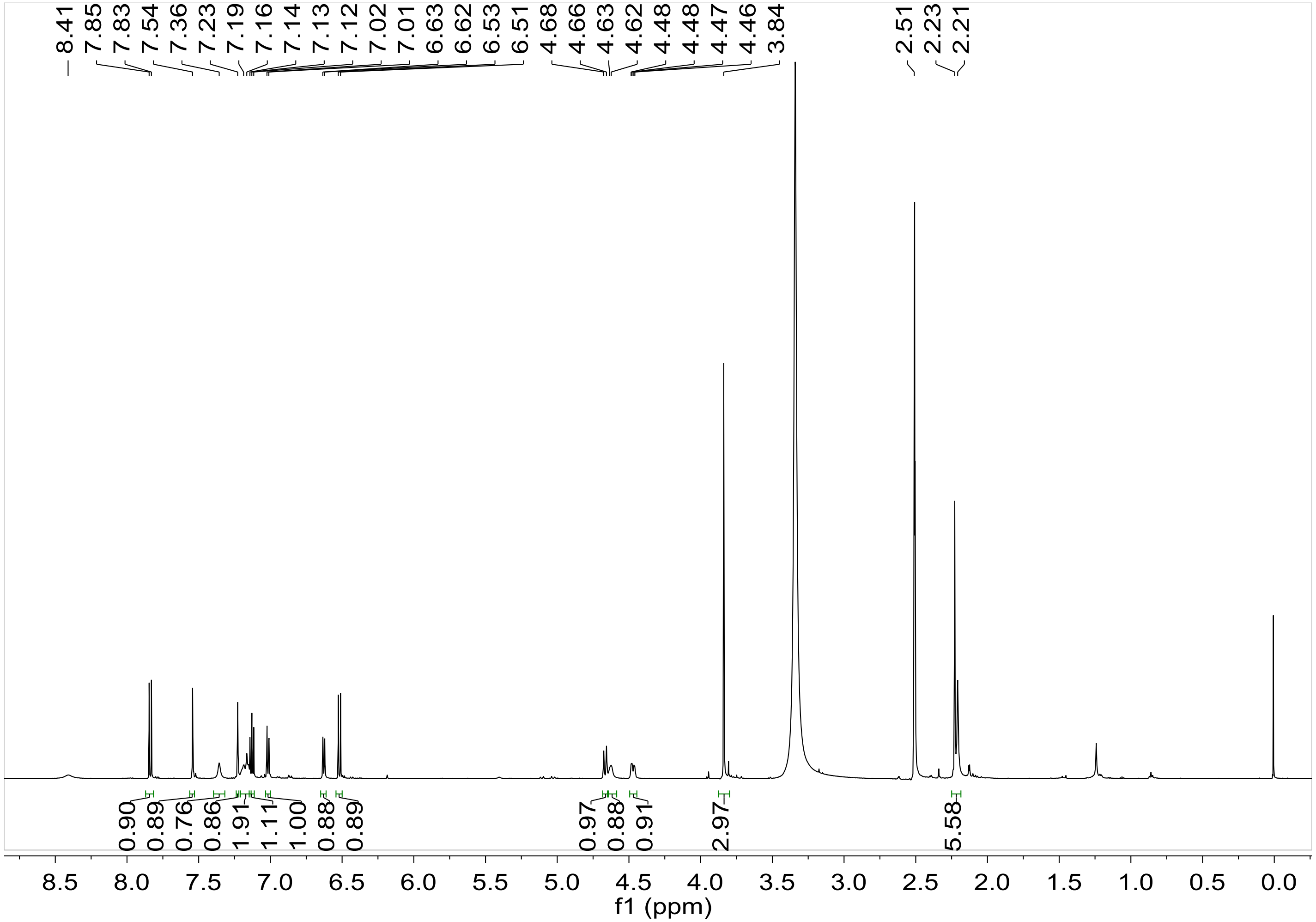

Figure S7. The ^{13}C NMR (150 MHz) spectrum of **4** in acetone-d_6.

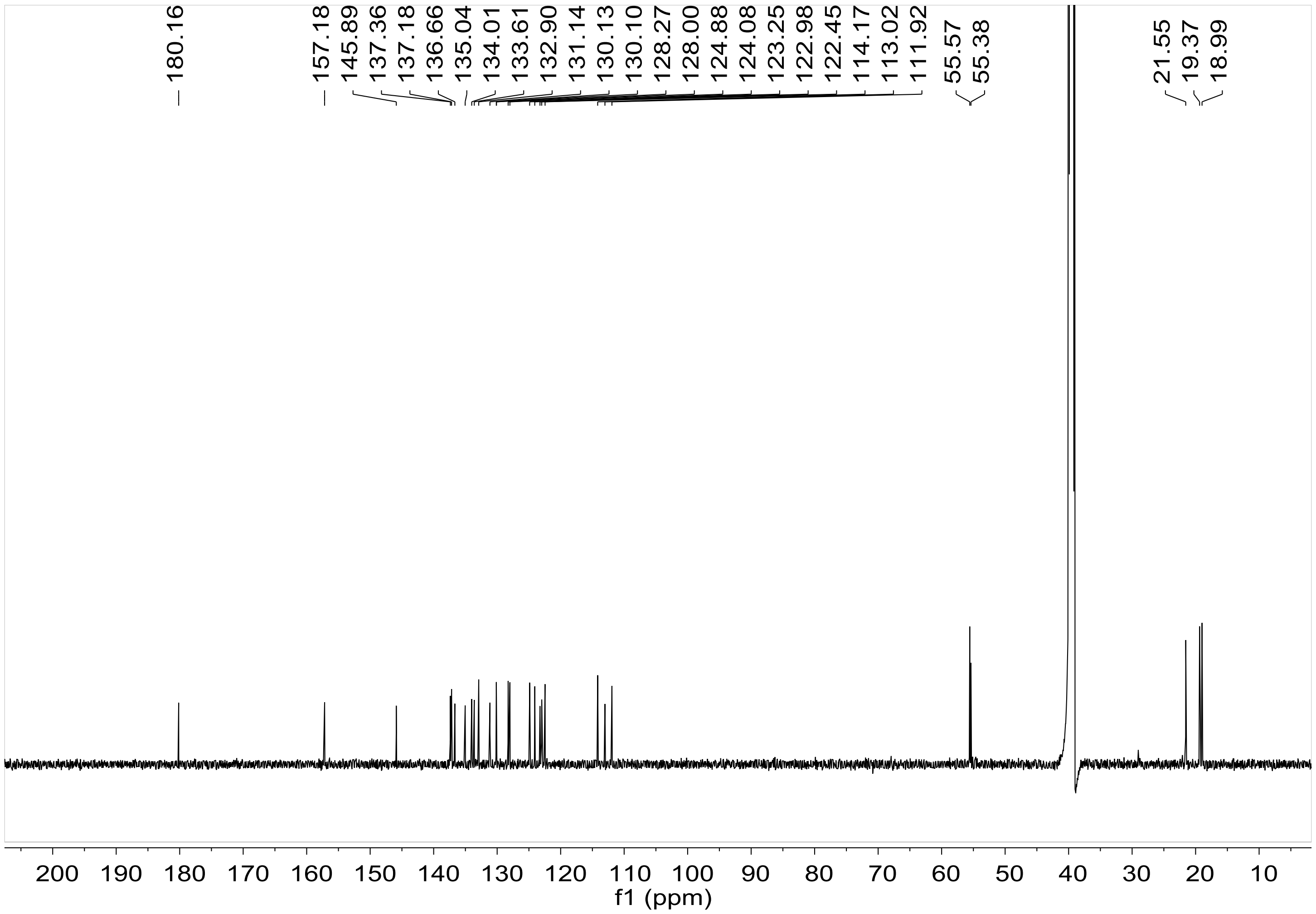

Figure S8. The HRESIMS spectrum of **4**.

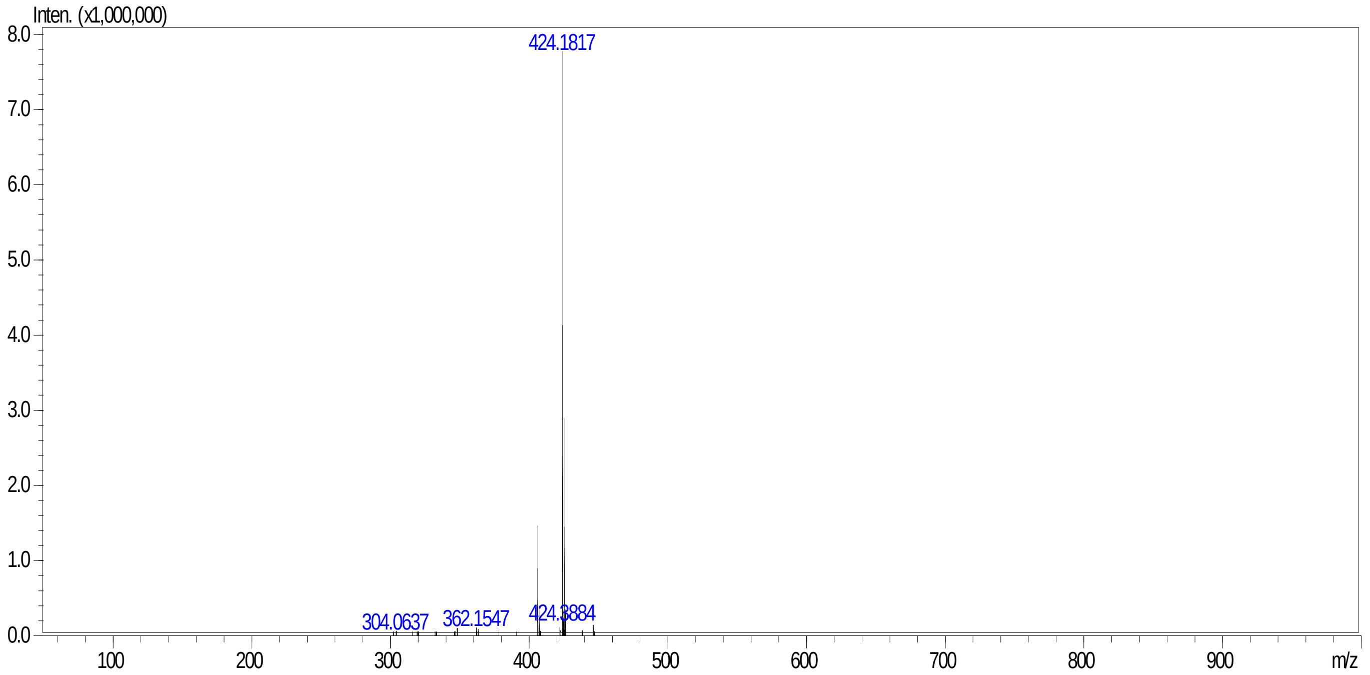

Figure S9. The IR spectrum of **4**.

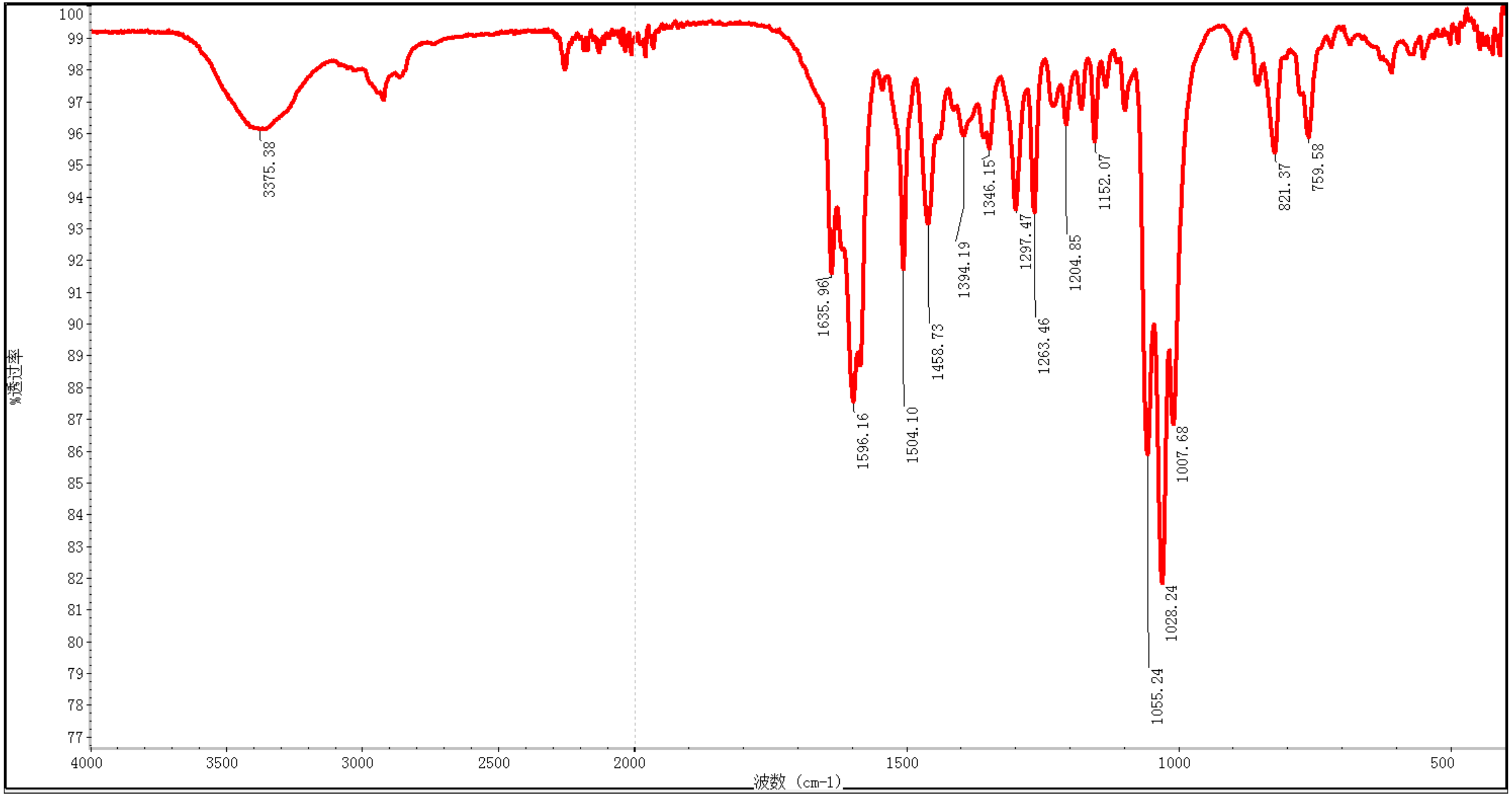

Figure S10. The ^{1}H NMR (600 MHz) spectrum of **5** in DMSO-d_6.

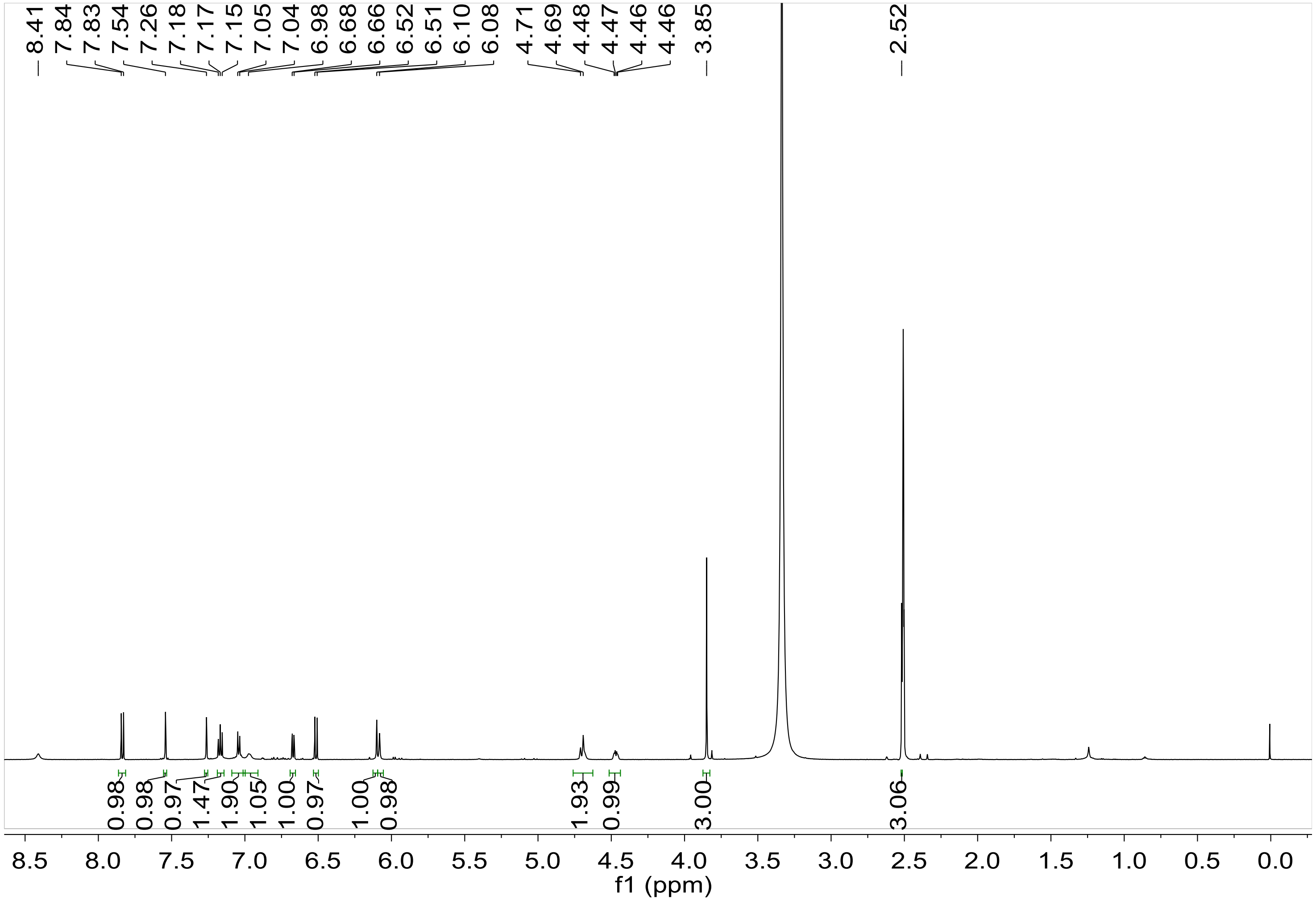

Figure S11. The ^{13}C NMR (150 MHz) spectrum of **5** in DMSO-d_6.

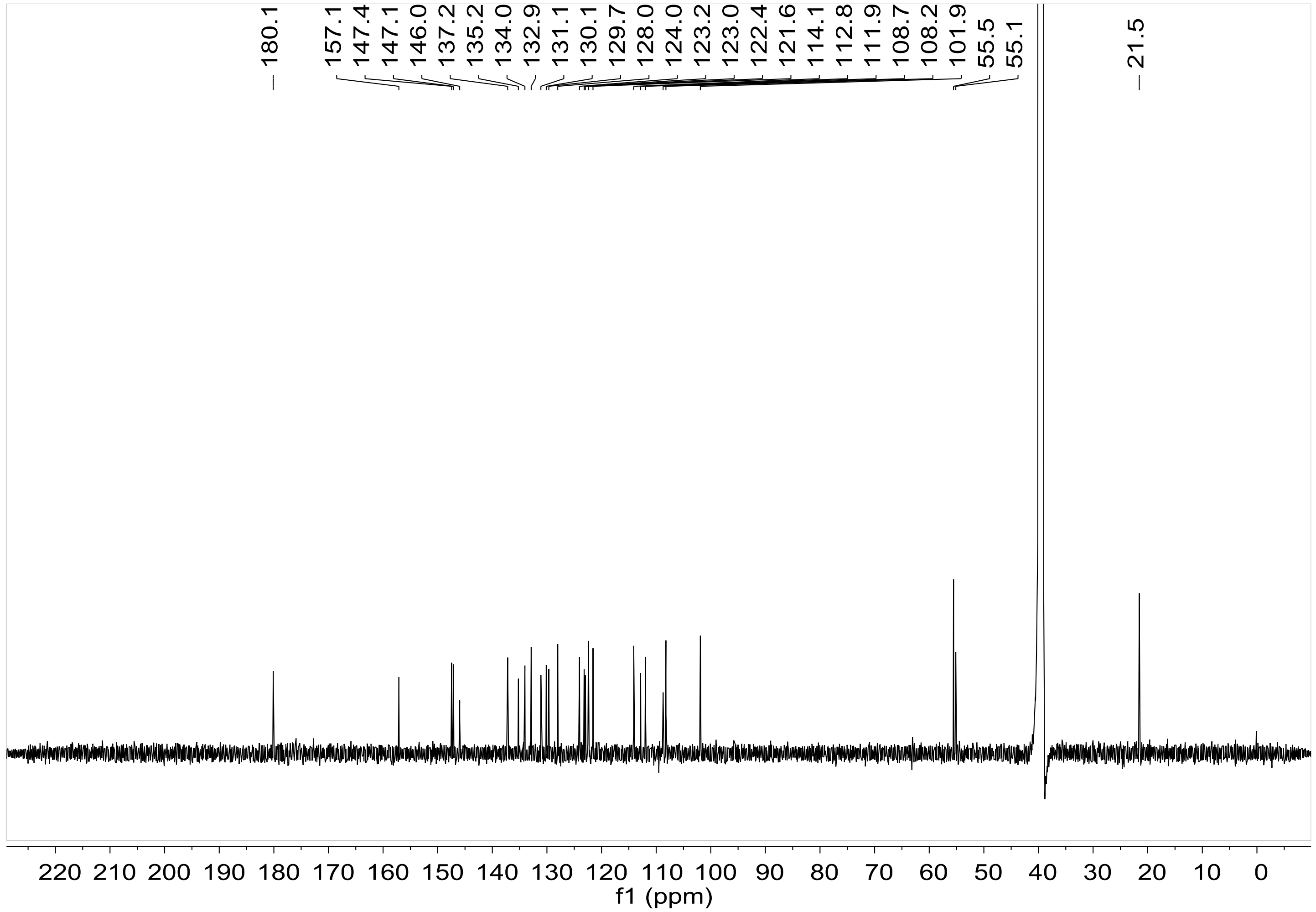

Figure S12. The HRESIMS spectrum of **5**.

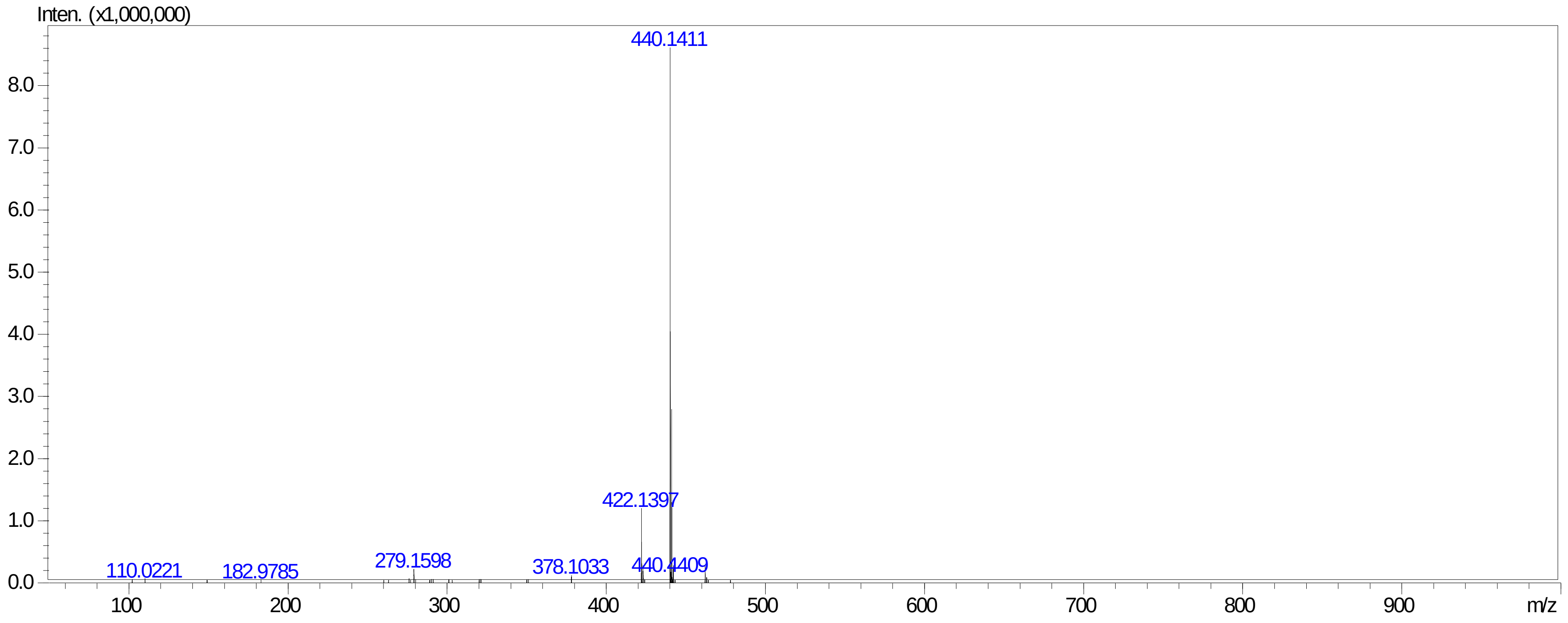

Figure S13. The IR spectrum of **5**.

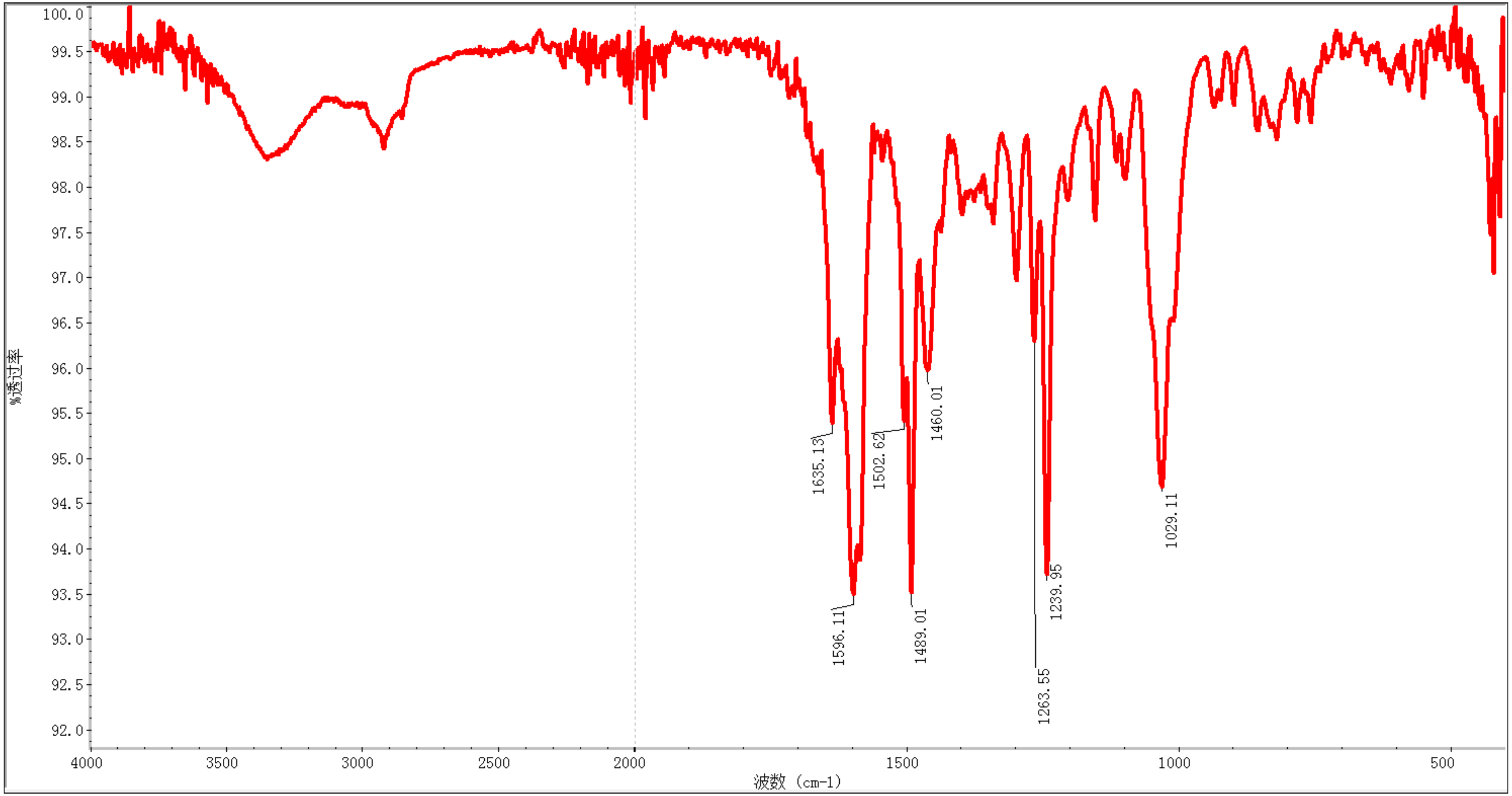

Figure S14. The ^{1}H NMR (600 MHz) spectrum of **6** in DMSO-d_6.

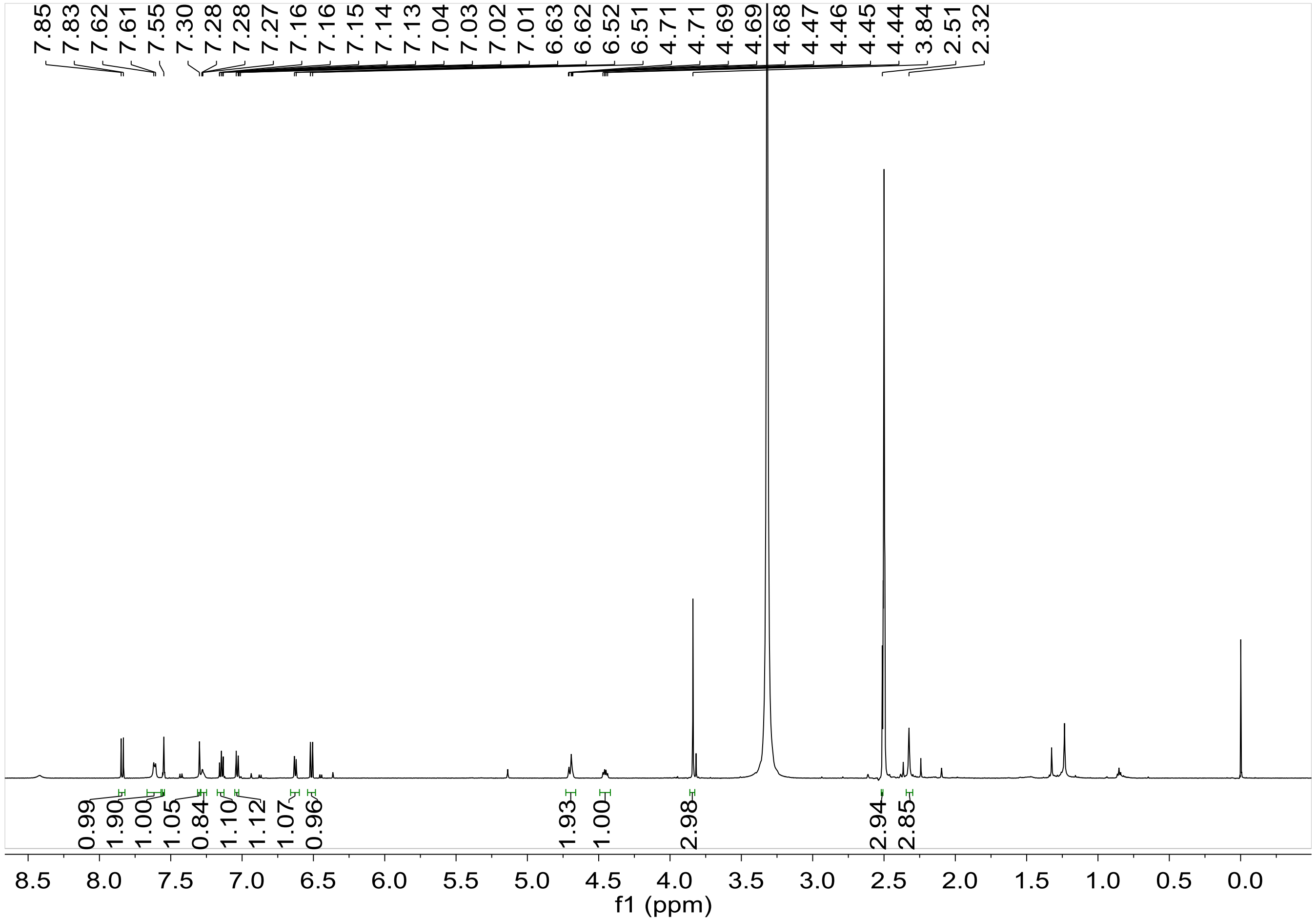

Figure S15. The ^{13}C NMR (150 MHz) spectrum of **6** in DMSO-d_6.

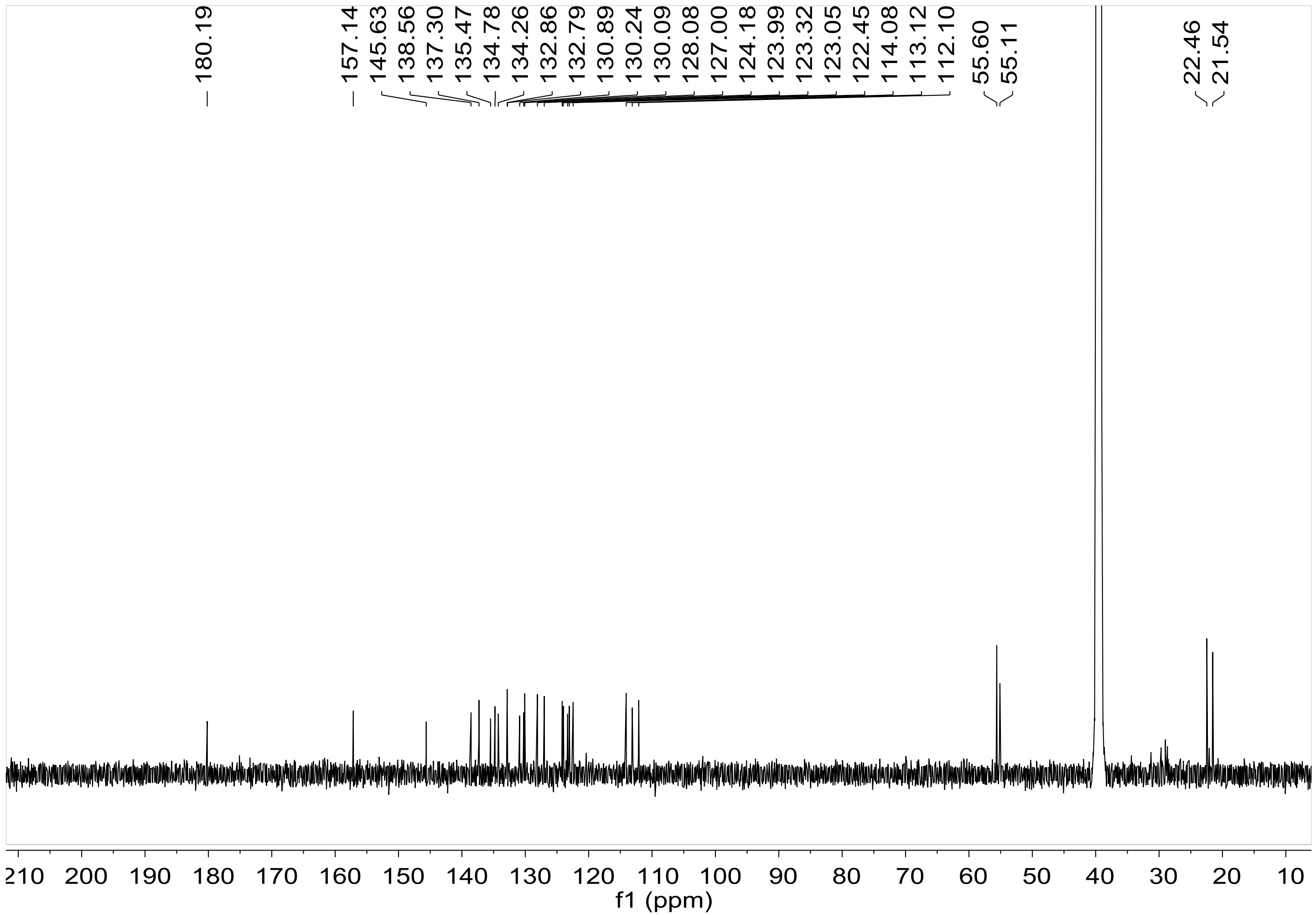

Figure S16. The HRESIMS spectrum of **6**.

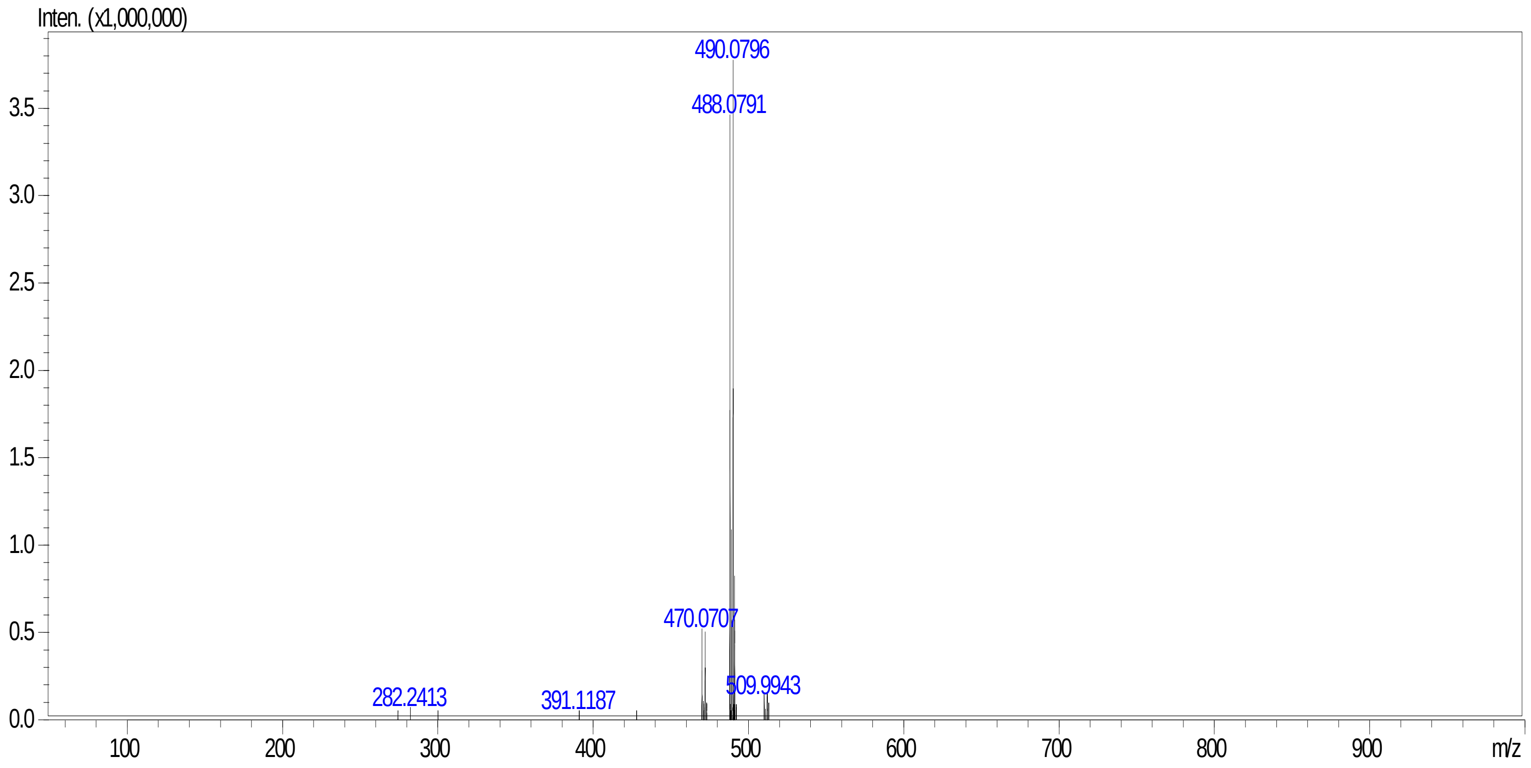

Figure S17. The IR spectrum of **6**.

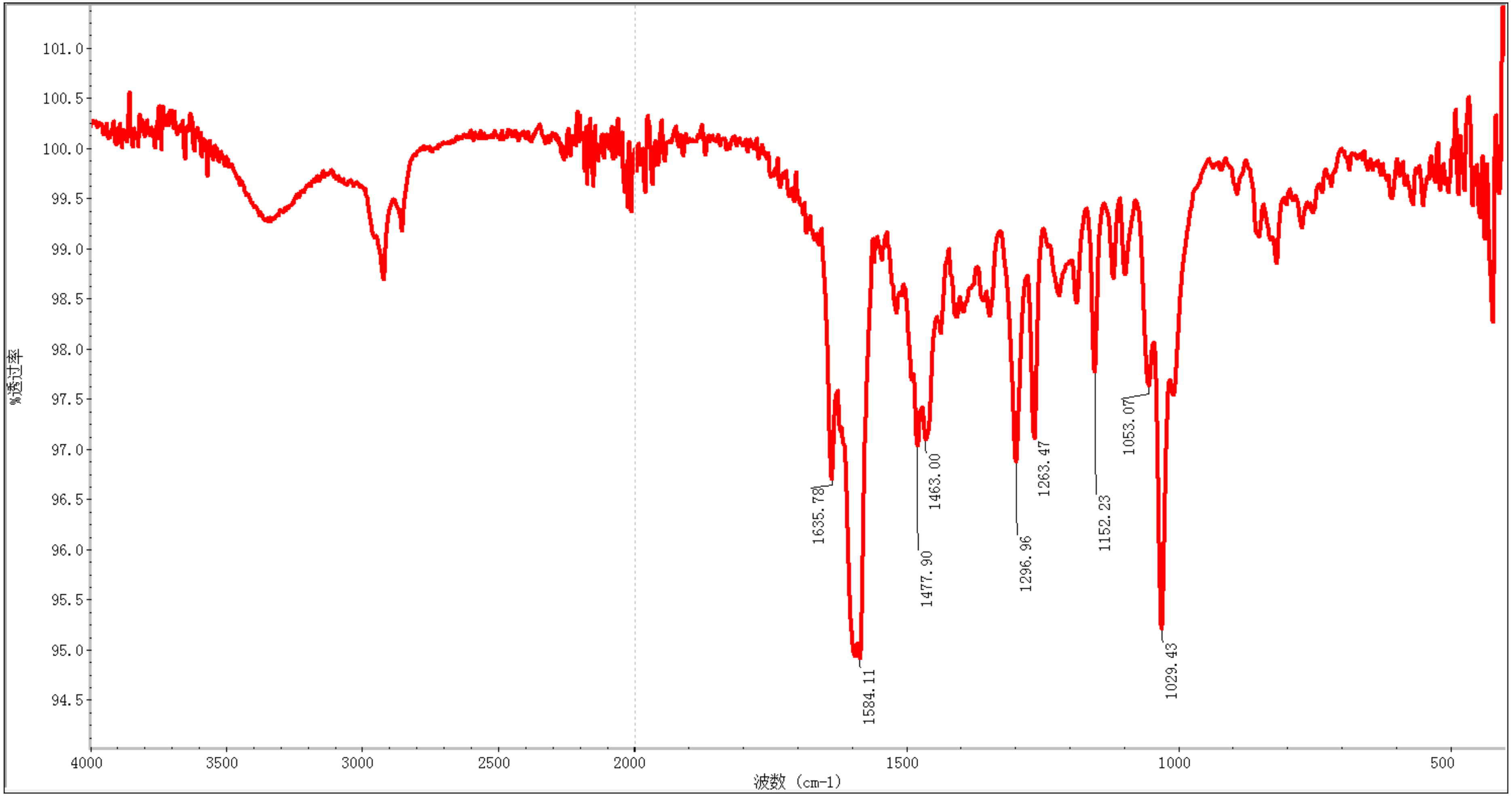

Supplement: Supplementary file 1 [file molecules-26-01959-s001.pdf]
